# Extreme heat and hospitalization with Parkinson’s disease among older adults

**DOI:** 10.1038/s41370-026-00882-7

**Published:** 2026-04-13

**Authors:** Claire Dinehart, Scott W. Delaney, Lauren Mock, Brad A. Racette, Gary W. Miller, Marianthi-Anna Kioumourtzoglou, Danielle Braun, Antonella Zanobetti, Daniel Mork

**Affiliations:** 1 Department of Environmental Health, Harvard T.H. Chan School of Public Health, Boston, MA, USA.; 2 Department of Biostatistics, Harvard T.H. Chan School of Public Health, Boston, MA, USA.; 3 Barrow Neurological Institute, Phoenix, AZ, USA.; 4 Department of Environmental Health Sciences, Mailman School of Public Health, Columbia University, New York, NY, USA.; 5 Department of Epidemiology, School of Public Health, Brown University, Providence, RI, USA.; 6 Institute at Brown for Environment and Society, Brown University, Providence, RI, USA.; 7 Department of Data Science, Dana-Farber Cancer Institute, Boston, MA, USA.

**Keywords:** Climate change, Health studies, Epidemiology

## Abstract

**BACKGROUND:**

The projected rise in U.S. Parkinson’s Disease (PD) cases makes understanding risk factors crucial to plan for changing healthcare utilization patterns. Factors such as extreme heat exacerbated by shifts in regional climate conditions may present additional risks among the PD population.

**OBJECTIVE:**

We aim to determine whether nationwide and climate region-specific acute heat index exposure is associated with increased rates of hospitalizations with PD among the Medicare population.

**METHODS:**

We follow a time-stratified case-crossover study design for a population of Medicare fee-for-service beneficiaries within the contiguous U.S. hospitalized between 1 January 2000 and 31 December 2016. The exposure of interest is the daily maximum heat index during the warm season (May–September), converted to percentiles using climate region-specific warm season heat index distributions. We used distributed lag models to estimate the immediate and lagged associations of heat index on hospitalization with PD.

**RESULTS:**

Our sample included 427,813 individuals (89.3% White, 47.7% female, mean age 79.8). In nationwide analyses, the odds ratio (OR) of hospitalization with PD comparing days in the 99th versus 50th percentile of the heat index distribution was 1.010 (95% CI 1.002, 1.017). Results suggest extreme heat effects persist 2 days beyond the initial day. The cumulative OR of hospitalization with PD after 3 days of continuous exposure (i.e., cumulative over lags 0–2) to heat indexes at the 99th versus 50th percentile was 1.022 (95% CI: 1.005, 1.039). Estimates were larger in temperate climates, while tropical, arid, and continental regions showed varying impacts with non-significant associations.

**SIGNIFICANCE:**

High heat index exposure is associated with increased odds of hospitalization with PD amongst older adults, particularly those living in temperate climates in the US South. These results may inform both medical practice and policy through crafting PD-patient-centered hotweather advice and via the issuance of local and state extreme heat advisories.

**IMPACT:**

For an aging society, the threat of increased frequency of extreme heat from climate change poses significant risks to older adults, especially those living with Parkinson’s Disease (PD). Our work addresses gaps in several preceding studies by exploring more fully how effects of extreme heat on the risk of PD-related hospitalization differ across climate types and by sex. The additional insight presented in our analysis can equip practitioners and policymakers with the data required to help mitigate the threats of extreme heat on patients with PD.

## INTRODUCTION

Parkinson’s disease (PD) is the second most common neurodegenerative disease globally [1]. PD risk increases with age, with an estimated incidence rate of 108–212 per 100,000 adults age 65 years and older in the United States (U.S.) [2]. In 2017, over 1 million U.S. residents were living with a PD diagnosis. By 2037, this number is expected to rise to 1.6 million as the U.S. population ages [3]. Thus, understanding risk factors for hospitalization with PD in an aging population is crucial for adults living with the disease, clinicians managing the disease, and policymakers seeking to mitigate the public health burden of the disease. In our study, we focus on hospitalizations, which provide a particularly salient proxy for heat-related morbidity because they indicate a need for heightened or prolonged medical care.

One possible risk factor for hospitalization with PD may be extreme heat and prolonged periods of increased heat, which have become a rapidly increasing public health concern due to rising global temperatures associated with climate change [4–10]. Adults with PD have been shown to be particularly susceptible to extreme heat [4]. For example, adults with PD are at higher risk of oxidative stress due to the exacerbation of PD-related mitochondrial dysfunction from exposure to high temperature and humidity [11]. Pro-inflammatory effects of elevated temperature exposure may also exacerbate the systemic inflammation associated with PD [12, 13]. Other autonomic dysfunction common to PD, including catecholaminergic dysfunction altering cardiovascular function or sweating abnormalities (including both hypo- and hyperhidrosis), impacting thermoregulation, are contributors to heightened heat susceptibility in this population [14–16]. For example, a PD patient’s orthostatic hypotension could be exacerbated by dehydration from increased temperatures [17].

Nevertheless, prior research investigating relationships between extreme heat and PD-related outcomes is limited. Early studies report that exposure to multiple measures of increased temperature is associated with adverse PD-related health outcomes [18–22]. For example, Linares et al. reported that higher daily maximum temperature was associated with increased PD-related emergency hospitalizations in Madrid, Spain [22]. However, the Linares et al. study was from a climatologically homogenous location and is limited in its generalizability to other climates and countries.

Health effects from extreme heat can vary widely across climate types [23, 24]. Individuals adapt and acclimatize to their specific climate. For example, in hot climates, these may include earlier sweating, preservation of electrolytes, and increased skin blood flow [25]. Infrastructure differences between regions and countries can also modify these effects [23]. Thus, it remains unclear how extreme heat may impact the risk of hospitalization with PD in the United States or among its different climate regions. Moreover, while prior studies generally measure daily temperature, humidity may combine with temperature to alter effects [26, 27]. Finally, prior evidence suggests that both PD symptoms and effects of heat may differ by sex [28–32]. But to the best of our knowledge, no prior studies have explored how the effects of extreme heat on hospitalization among adults with PD may differ between women and men.

Our study builds on previous work by extending analyses to the entire contiguous U.S. and stratifying by climate region to address the potential impacts of acclimatization to heat conditions within each region. We considered heat index as the exposure of interest, rather than merely temperature, which combines both temperature and humidity as a better measure of how current outdoor conditions impact the human body. Higher humidity and temperature correspond to higher heat index values. In this study, we used a time-stratified case-crossover design to examine the risk of hospitalization with PD among Medicare beneficiaries from 2000–2016 during warm-season months (May–September). We hypothesized that exposure to higher heat index values would lead to a greater risk of hospitalization with PD, and we used distributed lag models to examine the most important windows of susceptibility, with potential for variation by climate type.

## METHODS

### Study population and outcome

The study population was drawn from a cohort of Medicare fee-for-service (FFS) beneficiaries aged 65+ within the contiguous United States (Alaska, Hawaii, and U.S. territories excluded) who experienced a hospitalization between 1 January 2000 and 31 December 2016. We defined any hospitalization as a “case” if its resulting claim included a PD-related ICD-9 (332.0) or ICD-10 (G20) code within the first ten billing diagnosis codes. We included only the first such hospitalization for each beneficiary, resulting in a cohort of unique individuals. To capture hospitalizations in the warm season, the population was restricted to first hospitalizations between May 1st and September 30th. Because we sought to examine effects of extreme heat among community-dwelling Medicare beneficiaries—and not among beneficiaries living in skilled nursing facilities who were less likely to be exposed to extreme outdoor temperatures—we limited our sample to cases in which the beneficiary was admitted either (1) from a non-health care facility point of origin, or (2) via a clinic referral; or (3) via the emergency room. Participants were excluded based on unavailable daily exposure data, including temperature, humidity, and air pollution.

Individual demographics, including age, sex, racial and ethnic identity, dual eligibility for Medicaid (a proxy for individual-level socioeconomic position), and residential ZIP code, were sourced from Medicare data.

### Exposure

Daily maximum temperature and daily minimum relative humidity were obtained from the Gridded Surface Meteorological (gridMET) data with a gridded resolution of 4 km by 4 km [33]. The exposure of interest was the warm season daily maximum heat index (1st May–30th September), a metric that relays the synergistic impacts of temperature and humidity [27]. The heat index was calculated using the “weathermetrics” package in R using an algorithm established by the U.S. National Weather Service [27]. We assigned heat index values to beneficiaries based on their residential ZIP code.

Following Delaney et al., we assigned beneficiaries to Köppen-Geiger climate types based on their residential county [34, 35]. Köppen-Geiger climate types classify geographies based on seasonal temperature and precipitation patterns. There are 4 Köppen-Geiger climate types represented in the contiguous United States (arid, continental, temperate, tropical), consisting of 18 subtypes. To address acclimatization and adaptation as well as differing distributions of heat across climate subtypes, we transformed heat index values into percentiles based on the observed heat index values within each climate subtype. We performed a nationwide analysis and stratified analyses by climate type.

Because air pollution has been shown to be associated with hospitalization with PD, we linked daily air pollution (PM_2.5_, NO_2_, and ozone) data to ZIP codes using well-validated air pollution models described elsewhere for use in secondary models as a sensitivity analysis [36–40]. However, high ambient temperatures may cause higher air pollution levels, and these sensitivity models adjusting for air pollutants estimate the direct effect of extreme heat on hospitalization with PD after accounting for any indirect effect of extreme heat that is mediated by increased air pollution [41].

### Statistical analysis

The association between heat index exposure and first hospitalization with PD was examined with a time-stratified case-crossover design [42, 43]. A case-crossover study is a variation of a case-control design, in which participants serve as their own control and are adjusted for time-invariant confounding risk factors (e.g., chronic comorbidities or socioeconomic status). Case days were determined as the day of the first hospitalization with PD. Control days were matched on the same year, month, and day of the week (e.g., Monday, Tuesday, etc.) as the case day. For example, if a first hospitalization with PD occurred on Tuesday, July 15, 2014, then the corresponding control days would be Tuesday, July 1st, 8th, 22nd, and 29th of 2014.

We fit conditional logistic regression models to estimate the association between heat index and hospitalization with PD. For each case or control day, we linked the same-day (lag day 0) and previous 14 days’ (lag days 1–14) heat index values, and we estimated time-lagged effects of heat index exposures on odds for hospitalization using constrained distributed lag nonlinear models (DLNMs) [44]. We fit DLNMs using cubic regression splines with degrees of freedom and knot placements based on Akaike information criterion (AIC) values, which measure how well each model fits the underlying data. Specifically, to regularize the model due to the high degree of day-to-day autocorrelation in heat index exposures we allowed for 3–5 degrees of freedom for the lag dimension with knots equally spaced on a log scale; and we fixed 3 degrees of freedom in the exposure dimension and specified that the center knot be placed at one of the following percentiles: 70th, 75th, 80th, 85th, or 90th. We fit models that included all beneficiaries regardless of climate type (“nationwide model”) and models stratified by climate type (AIC selected degrees of freedom and knot placement for each model are described in Supplementary Table 1). We also considered nationwide and climate-type specific models stratified by sex because prior research suggests sex differences in PD disease progression and symptom severity. As a supplemental analysis, we additionally stratify the national-scale model by race. Throughout the results, we use α = 0.05 as the level of significance and do not adjust for multiple testing. Software code to reproduce all analyses is available at: https://github.com/NSAPH-Projects/AcuteTemp_PDhosp.

## RESULTS

**Fig. 1:**
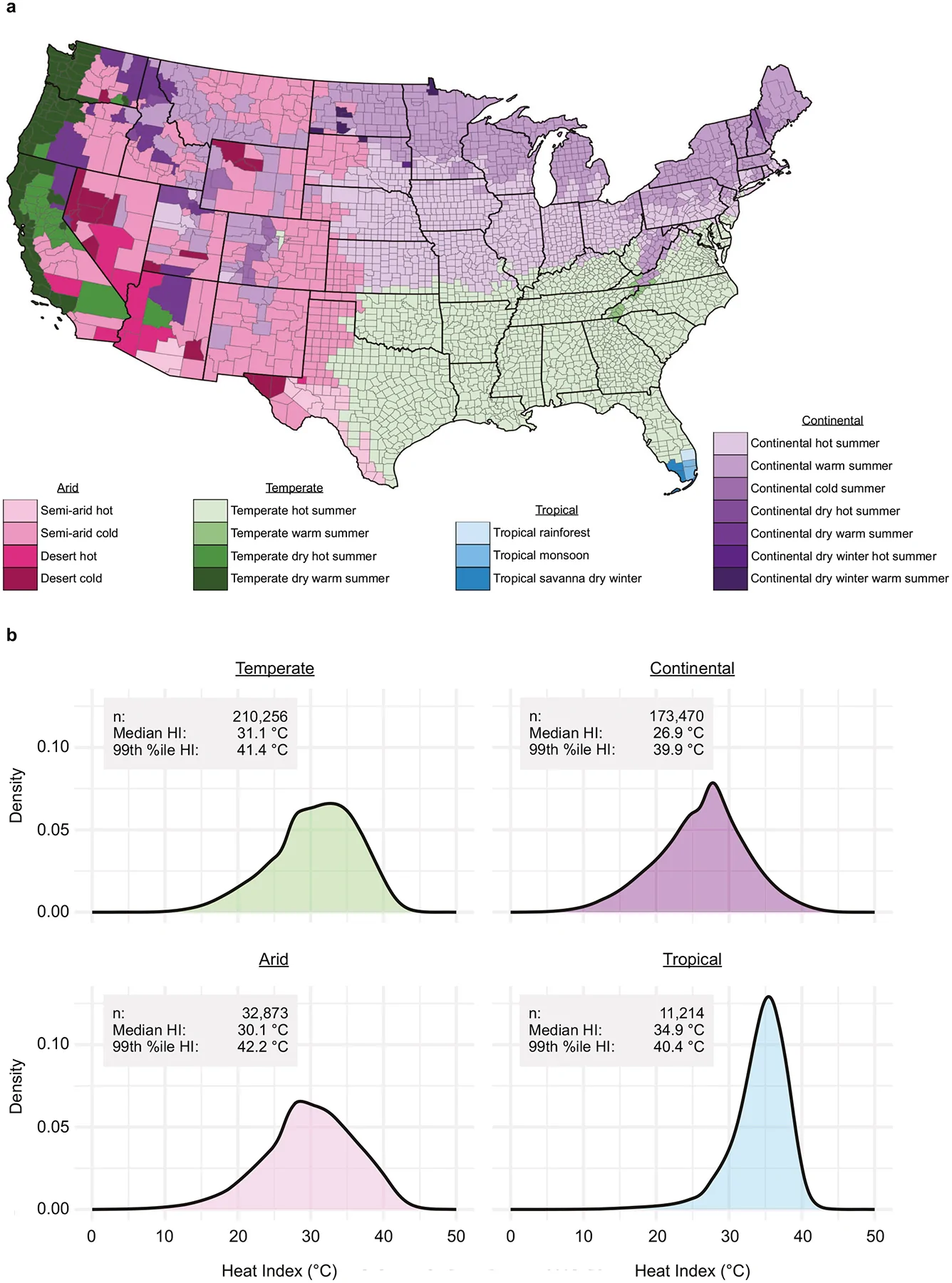
Köppen-Geiger climate types and warm-season heat index distributions in the United States. Shown in **a** are the 4 climate types and 18 climate subtypes represented in the contiguous United States by county. **b** shows distributions of daily maximum heat index values for all case and control days within each climate type during the warm season (May-September) during the period from 2000 to 2016. “*n*” values are total hospitalizations with PD (i.e., cases) in each climate type in our sample.

Our study included 427,813 unique individuals with first hospitalizations with PD amongst Medicare beneficiaries during the warm season from 2000 to 2016 in the contiguous U.S. Demographic information are displayed in Table 1 both nationally and stratified by climate region; most participants across all climate regions were White (89.3%), male (52.3%), not Medicaid eligible (84.6%), with an average age ranging from 79 to 81 depending on climate region. Median and extreme heat index values differed by climate (Fig. 1).

**Fig. 2:**
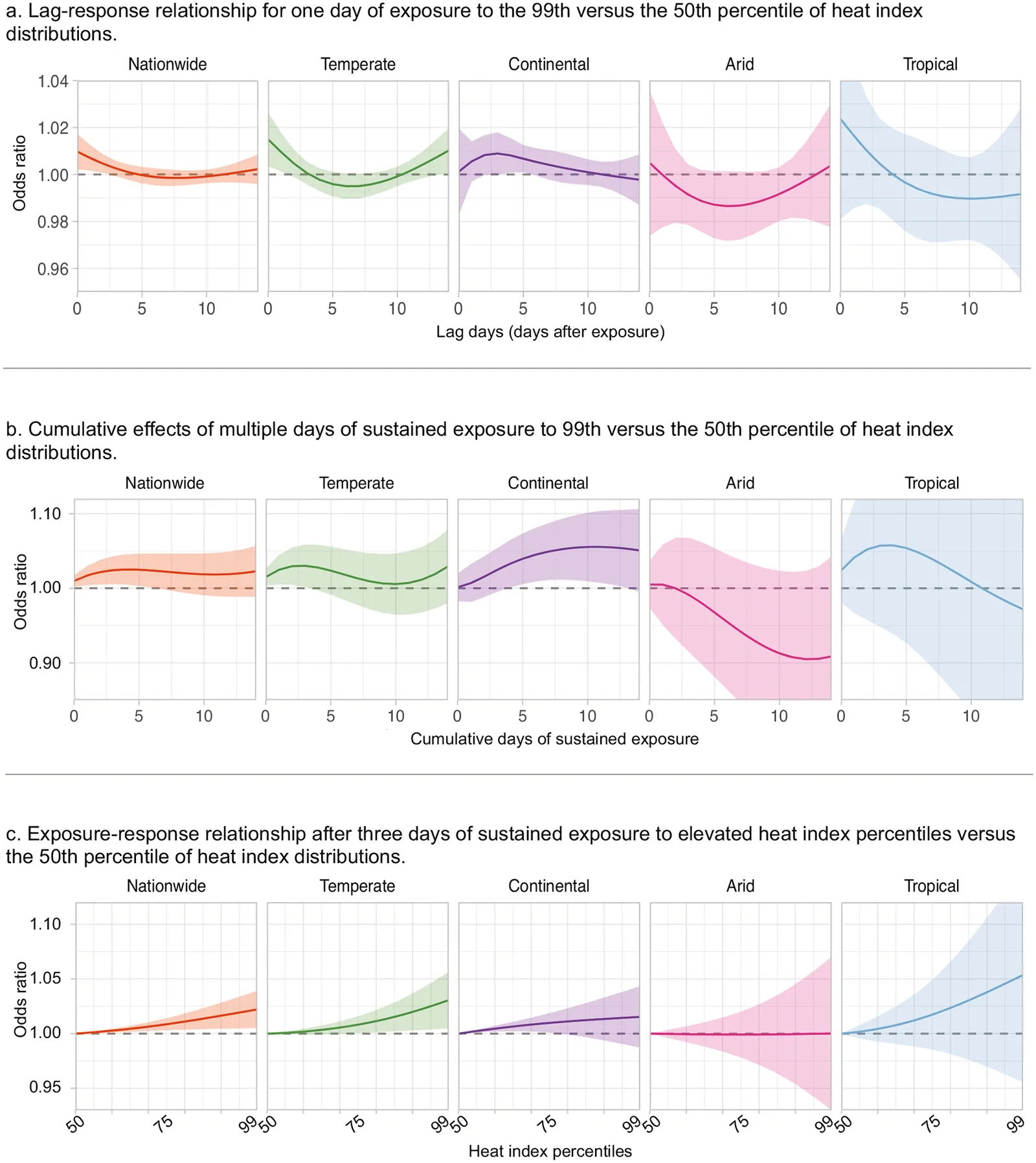
Time-lagged and cumulative risk of hospitalization with PD from extreme heat exposure. **a** shows the lag-response relationship from the distributed lag model representing the odds ratio of hospitalization with PD at the 99th versus the 50th percentile of the heat index distribution persists for up to 2 days thereafter before returning to baseline levels (Supplementary Table 2). **b** shows how total hospitalization risk accumulates (i.e., combining ORs across days, beginning from day 0, from (**a**)) due to sustained exposure to heat indexes in the 99th percentile versus the 50th percentile. Finally, **c** plots cumulative effects of 3 days of sustained exposure (i.e., combined ORs from lag days 0 to 2) to a range of climate-type specific heat index percentiles (compared to the 50th percentile), demonstrating that lower heat index values also confer additional risk of hospitalization with PD.

In nationwide analyses, we found that exposure to high percentiles of heat index were associated with a small increase in hospitalizations with PD on the day of extreme heat exposure as well as on the following two days (Fig. 2a). Thus, while we present the results for all lags in Fig. 2a (see below), we center much of our results and interpretations around the recent three days (i.e., lags 0–2) of exposure.

Figure 2a shows the effects of heat on the odds ratio (OR) of hospitalization with PD for each lag day of heat exposure at the 99th percentile, relative to the 50th percentile, nationally and for each climate region. For nationwide analyses, and for tropical, arid, and temperate regions, the effects are largest in the first few days before decreasing towards the null. For the continental region, effects are initially small, peaking in the fourth day before returning to an OR of approximately 1.0. In nationwide analyses, Medicare beneficiaries had an OR of 1.010 (95% CI 1.002, 1.017) for hospitalization with PD on the same day of heat index exposure at the 99th vs. 50th percentile, which attenuated to an OR of 1.007 (95% CI 1.002, 1.013) for exposure 1 day prior and 1.005 (95% CI 1.001, 1.009) at 2 days lag (Supplementary Table 2). Additional lag-specific ORs for the first three lag days of heat exposure at the 95th and 99th percentiles across climate regions are in Supplementary Table 2.

In nationwide analyses, Medicare beneficiaries had an OR of 1.022 (95% CI 1.005, 1.039) for a hospitalization with PD following 3 days (i.e., cumulative across lag days 0–2) of sustained heat index exposure at the 99th vs. 50th percentile (Table 2). When comparing across climate types, exposure to the 99th percentile (vs. 50th percentile) heat index in temperate regions had the strongest effect, with an OR of 1.030 (95% CI 1.004, 1.056) for a hospitalization with PD following sustained exposure to a heat index for three continuous days. Tropical, arid, and continental regions showed varying impacts, although the confidence intervals largely spanned the null and did not result in statistically significant associations. Table 2 presents estimated cumulative ORs for one, two, or three consecutive days of sustained heat exposure at the 95th and 99th vs. 50th percentiles. Figure 2b visualizes the cumulative effects of sustained heat exposure at the 99th vs. 50th percentile over a 14-day period.

**Fig. 3:**
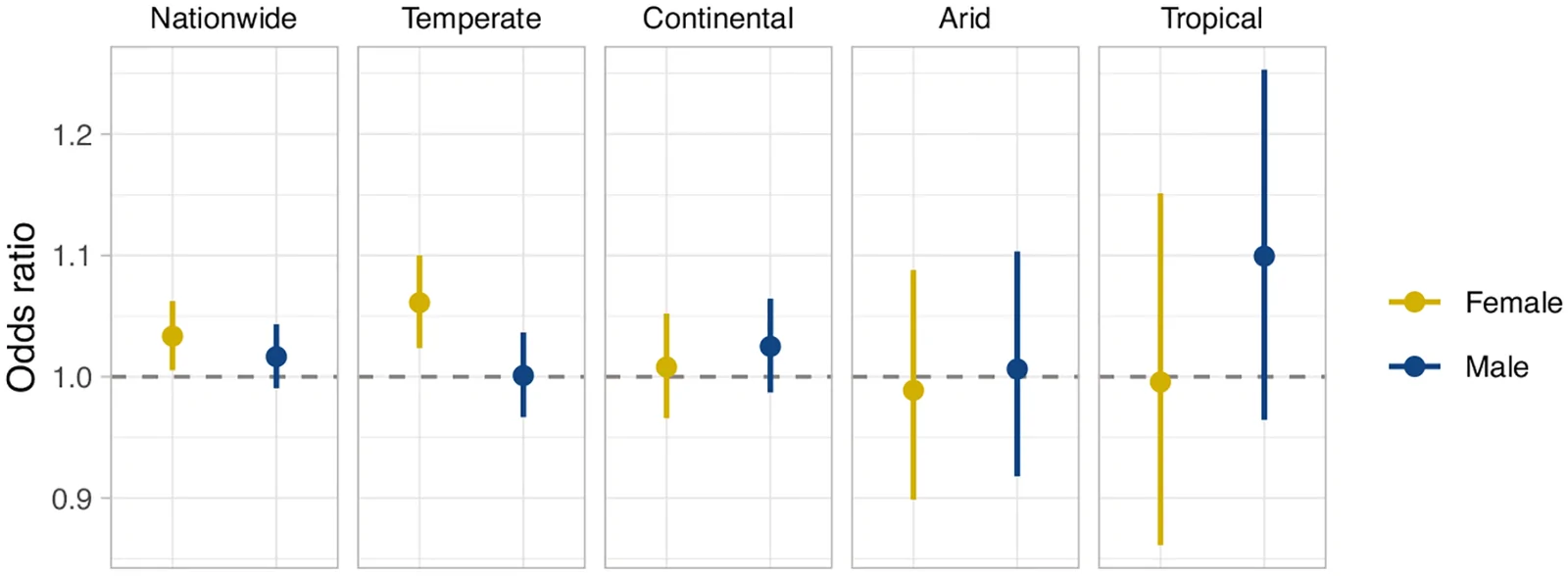
Odds ratios of hospitalization with PD after sustained exposure to 3 days of extreme heat, by climate and sex. Shown are the cumulative odds ratios and confidence intervals for hospitalization with PD after sustained exposure to 3 days (i.e., combining ORs across lag days 0–2) at the 99th versus the 50th percentile of the warm season (May–September) daily maximum heat index distribution. The solid red line indicates a null effect, i.e., OR = 1.00. See Supplementary Table 4 sex-specific OR.

Sex-stratified results revealed inconsistent patterns (Fig. 3). For example, in temperate regions, female (versus male) beneficiaries had a higher risk of hospitalization with PD after 3 days of sustained extreme heat exposure: OR_female_ = 1.061 (95% CI 1.024, 1.100); OR_male_ = 1.001 (95% CI 0.967, 1.037) (Supplementary Table 4). However, results from continental and arid climates did not suggest sex-based differences, and estimates from tropical climates were highly uncertain. Overall, in nationwide analyses, results suggest female beneficiaries may have a somewhat lower odds ratio of hospitalization with PD on the first day of extreme heat exposure but a greater cumulative risk than male beneficiaries for the first 3 days (Supplementary Table 4).

Results of the sensitivity analyses, additionally adjusting for air pollution, were largely unchanged (Supplementary Table 3). In a few instances, we found a small attenuation of the effects of heat index on hospitalizations with PD. However, even after adjusting for air pollution measures, we found associations between heat index and hospitalization with PD in both temperate regions and nationally remained significant, while estimates in tropical, continental, and arid regions were non-significant but of similar effect sizes as models not adjusted for air pollution. Stratifying by race, we found a similar lag-response shape for each subgroup (Supplementary Fig. 1) but larger magnitude effects among Black, Asian/PI, Hispanic, and Other subgroups; small sample sizes within subgroups resulted in larger confidence intervals often crossing the null (Supplementary Materials Table 5).

## DISCUSSION

This study examined the association between the immediate and short-term effects of warm-season heat index exposure and first hospitalization with PD amongst Medicare beneficiaries in the contiguous U.S. We found evidence that exposure to heat index values at the 95th and 99th percentiles (versus the 50th percentile) during the immediate and previous 2 days (i.e., lag days 0–2) was associated with an increased risk of hospitalization with PD. For association by climate type, temperate and tropical regions had larger effects relative to arid and continental regions, yet only the temperate region showed statistically significant associations. The small number of tropical regions in the continental US likely contributed to wider confidence intervals. We also conducted sensitivity analyses to consider the potential confounding effects of air pollutants on the heat index-hospitalization relationship, finding results similar to our main analyses.

While there is strong evidence regarding issues with temperature regulation in patients with PD, there are few epidemiological studies documenting the effects of extreme heat. We add to this knowledge base and note the need for more extensive work to quantify and understand the impacts of extreme heat on this vulnerable population. Our results build upon and substantially advance earlier, related research. Linares et al. reported results from Madrid, Spain, that were similar to our study, with positive associations between individual lag day and cumulative lagged heat exposures and hospitalization with PD [22]. Separately, Guirguis et al. [45] conducted a San Diego, California-based study on all-cause hospitalizations and found significant differences in all-cause hospitalization risk from extreme heat within San Diego County’s differing local climates [45]. A similar study on heat index and hospitalization with Alzheimer’s disease and related dementias found that effects due to extreme heat persisted for up to 4 days, with significant effects across temperate, continental, and arid climates, and some evidence that risks were larger for men than for women [34]. Similarly, our results also suggest differences by climate type, in which effects appear greater in temperate regions. Differing socioeconomic and demographic makeup between regions may contribute to these differences. However, the temperate region’s relatively larger sample size in our study (210,256 cases) may indicate that analyses in other regions in our study were underpowered. Our secondary supplemental analysis identified larger effects among non-White subgroups; however, most confidence intervals were overlapping. While not the focus of this paper, more work is needed to replicate subgroup differences and understand the factors driving increased risks.

We stratified by sex to evaluate potential differences in the effects of heat on hospitalization with PD. First day heat exposure in the temperate region was associated with 2–3% higher odds of hospitalization with PD in females in the 95th & 99th percentiles versus the 50th percentile of the heat index distribution. Examining the cumulative effect for three days of exposure, females had 5–6% higher odds of hospitalization with PD in the 95th & 99th (versus the 50th) percentiles. This is consistent with a research review and a Netherlands study that found sex differences in heat and all-cause mortality, where females aged 80+ had significantly greater risk of mortality compared to men [29, 30]. The risk mechanism for women vs. men is unclear, but theories include inefficient sweating and thermoregulation with age, the continuance of daily activities during high heat days, living alone, and the insulating effects of a higher fat percentage, but more research is needed to determine the factors under-pinning this possible difference [28].

In the tropical region, first-day heat exposure was associated with 4% higher odds of hospitalization with PD in males in the 95th & 99th percentiles. For three days of cumulative exposure, males had 8–9% higher odds of hospitalization with PD, but confidence intervals for this region were wider, though, reflecting the small sample size for the region. Previous studies on heat illnesses and emergency department visits have found a higher risk for men, indicating that the lack of significance in our study could be related to sample size and statistical power [46, 47].

Our study has some limitations. Outcome misclassification could result if hospital billing professionals did not indicate that a beneficiary with PD had PD in the first 10 diagnosis codes assigned to each hospitalization claim. This would likely bias our results toward null values, potentially underestimating the effects. However, ICD code reviews found no evidence of widespread under/over-reporting, with an average positive predictive value of 70% for PD [48]. Second, we used ambient measures of temperature, humidity, and air pollution, but these metrics are modeled, and heat index values may not precisely measure the heat index experienced by each beneficiary, e.g., our measures do not account for possible air conditioning use or other sources of indoor air pollution [49]. We use the heat index because it combines measures of temperature and humidity to more fully reflect the physiology of human body heat transfer [50]. However, heat index does not account for solar radiation or windspeed, which may further affect the experience of heat [51]. Third, assigning exposure percentiles based on Koppen-Geiger subregions may best approximate the distribution of temperatures experienced by each beneficiary, but subregions can be geographically large and therefore induce measurement error. While we stratified our analysis by Koppen-Geiger climate types to account for differences in acclimatization, each climate type may also entail heterogeneous built, social, and political environments. Future research should explore whether and how heat-PD associations differ by such factors in greater detail. Finally, the age of our cohort (65 years and over) is both a limitation and a strength. While we cannot speak on the impacts of heat on PD outcomes in younger age groups, PD typically presents in later life, indicating that our sample is generally representative of the population of interest [52]. Our study cohort includes only individuals who have been hospitalized with a PD diagnosis code, and results may not be generalizable to all Medicare beneficiaries with PD.

Our study has several strengths. Participant data are sourced from the largest available national U.S. cohort and involve 427,813 unique individuals. The case-crossover study design allowed for control of individual and area-level factors, including demographics, persistent environmental stressors, and other factors that do not vary within a month. Our results are comprehensive, accounting for the lagged effects of heat exposure across multiple days to estimate the compounding effects on hospitalization with PD. The high spatial and temporal resolution temperature and humidity data allow for more precise measurements of exposure for each individual in our study. Calculating climate subtype heat index percentiles allowed us to account for acclimatization to local climate and for varying distributions of exposure. Finally, we utilized flexible exposure-time-response models to allow for a nuanced characterization of the relationship between heat index, time of exposure, and hospitalization with PD.

In conclusion, we found that heat index exposure is associated with increased odds of hospitalization with PD amongst Medicare FFS beneficiaries. Additionally, factors like climate region and sex may alter the risks of hospitalization with PD amongst those aged 65 +. Our results may inform both policy and clinical practice. Policy implications include the issuance of local and state heat advisories, and PD support organizations may disseminate PD-specific guidance to warn patients of impending risk during periods of forecasted high heat. Additionally, medical facilities may consider releasing hot-weather advice specifically for older adults with PD to prevent excess hospitalizations during extreme heat events.

## Supplementary Material

supplement

**Supplementary information** The online version contains supplementary material available at https://doi.org/10.1038/s41370-026-00882-7.

## Figures and Tables

**Table 1.** Characteristics of Medicare beneficiaries aged 65 years and older who were hospitalized with a PD diagnosis code from 2000–2016.

	Nationwide		Climate type							
	(All climates combined)		Temperate		Continental		Arid		Tropical	
	Beneficiaries	Case day heat index	Beneficiaries	Case day heat index	Beneficiaries	Case day heat index	Beneficiaries	Case day heat index	Beneficiaries	Case day heat index
	[*n* or %]^a^	[mean (sd)]^b^	[*n* or %]	[mean (sd)]	[*n* or %]	[mean (sd)]	[*n* or %]	[mean (sd)]	[*n* or %]	[mean (sd)]
Total	4,27,813	28.9 (6.4)	2,10,256	30.6 (5.9)	1,73,470	26.3 (6.1)	32,873	29.9 (6.3)	11,214	34.2 (3.8)
Age										
65–74 yr	21.7	29.1 (6.3)	22.6	30.8 (5.8)	20.6	26.3 (6.0)	23.3	30.1 (6.3)	16.3	34.7 (3.4)
75–84 yr	46.2	28.9 (6.4)	46.4	30.6 (5.9)	46.2	26.2 (6.2)	46	29.8 (6.3)	43	34.3 (3.7)
≥85 yr	32.1	28.7 (6.4)	31	30.4 (6.0)	33.1	26.2 (6.2)	30.7	29.8 (6.4)	40.6	34 (4.1)
Sex										
Female	47.7	28.9 (6.4)	48.4	30.6 (5.8)	47.3	26.2 (6.1)	45.2	29.9 (6.3)	47.8	34.2 (3.9)
Male	52.3	28.8 (6.4)	51.6	30.5 (5.9)	52.7	26.3 (6.2)	54.8	29.8 (6.4)	52.2	34.3 (3.8)
Race/Ethnicity										
White	89.3	28.8 (6.4)	87.3	30.6 (5.9)	92.5	26.2 (6.1)	87.4	29.7 (6.4)	79.8	34.1 (4.0)
Black	6.2	29.6 (6.2)	8	31 (5.7)	5	26.5 (6.1)	1.6	29.4 (6.0)	4.4	34.6 (3.3)
Hispanic	2	31.4 (6.0)	1.6	31 (6.0)	0.8	26.8 (6.0)	6.3	32.8 (5.5)	14.2	34.6 (3.2)
Asian	1.2	27.4 (5.8)	1.6	27.5 (5.8)	0.6	26.8 (6.1)	1.9	28 (5.1)	0.4	34.8 (4.2)
Other	1.4	28.2 (6.1)	1.5	28.5 (6.1)	1.1	26.4 (5.9)	2.8	29.7 (5.6)	1.3	34.8 (3.3)
Medicaid Eligibility										
Eligible	15.4	29.4 (6.2)	17.9	30.3 (5.8)	10.9	26.3 (6.0)	19	30.8 (6.0)	26.5	34.1 (4.1)
Ineligible	84.6	28.8 (6.4)	82.1	30.7 (5.9)	89.1	26.3 (6.2)	81	29.6 (6.4)	73.5	34.7 (3.1)

a Percentages represent the percentage of beneficiaries with a given characteristic within the climate type listed at the top of the column. Thus, they are by climate type (column), and not by row.

b “Case day heat index” is the maximum heat index value in degrees Celsius estimated for the ZIP code in which a beneficiary resided on the day the beneficiary was hospitalized with a PD diagnosis code.

**Table 2.** Cumulative odds ratios of hospitalization with PD after 1 to 3 days of sustained exposure to the 95th and 99th percentile compared to the 50th percentile of the warm season^a^ daily maximum heat index distribution, nationwide and by climate.

	Days of sustained exposure^b^	Percentiles of the			
		climate-specific heat index distribution^c^			
		95th vs. 50th %ile		99th vs. 50th %ile	
Nationwide	1	1.009 (1.002, 1.015)	*	1.010 (1.002, 1.017)	*
(All climates	2	1.015 (1.004, 1.027)	*	1.017 (1.004, 1.030)	*
combined)	3	1.019 (1.005, 1.034)	*	1.022 (1.005, 1.039)	*
Temperate	1	1.013 (1.003, 1.023)	*	1.015 (1.003, 1.027)	*
climates	2	1.021 (1.004, 1.038)	*	1.025 (1.005, 1.045)	*
	3	1.025 (1.004, 1.047)	*	1.030 (1.004, 1.056)	*
Continental	1	1.002 (0.986, 1.018)		1.001 (0.983, 1.020)	
climates	2	1.007 (0.985, 1.030)		1.007 (0.981, 1.033)	
	3	1.014 (0.991, 1.039)		1.015 (0.988, 1.043)	
Arid	1	1.004 (0.978, 1.031)		1.005 (0.974, 1.037)	
climates	2	1.004 (0.960, 1.051)		1.005 (0.953, 1.060)	
	3	1.000 (0.944, 1.059)		1.000 (0.935, 1.070)	
Tropical	1	1.021 (0.983, 1.060)		1.024 (0.981, 1.069)	
climates	2	1.036 (0.971, 1.105)		1.041 (0.967, 1.121)	
	3	1.046 (0.963, 1.136)		1.052 (0.957, 1.157)	

a Warm season includes May-September of each year of the study period, 2000–2016.

b “Days of sustained exposure” are the number of consecutive days of exposure to the listed heat index. One day of sustained exposure corresponds to lag day 0 in distributed lag models. Because effects of extreme heat persist for up to 2 days past the initial day of exposure, effects accumulate as the duration of exposure increases.

c Starred (*) cells indicate 95% confidence intervals that do not include the null value.

## Data Availability

Exposure data is publicly available at https://www.climatologylab.org/gridmet.html and https://sedac.ciesin.columbia.edu/data/collection/aqdh/sets/browse. Medicare data is provided by the US Centers for Medicare and Medicaid Services; our data use agreement prohibits the sharing of the data sets used in our analysis. Academic and non-profit researchers interested in using Medicare data should contact the US Centers for Medicare and Medicaid Services directly.
