# Supplementary material for: Extreme heat and hospitalization with Parkinson’s disease among older adults
Source: J Expo Sci Environ Epidemiol. 2026 Apr 13;36(5):974–82. doi: 10.1038/s41370-026-00882-7 (PMC13335889; doi:10.1038/s41370-026-00882-7)
Supplement: Supplementary file 1 [file 41370_2026_882_MOESM1_ESM.docx]

**Extreme heat and hospitalization with Parkinson’s Disease among older adults**

Claire Dinehart, MS; Scott W. Delaney, ScD JD MPH; Lauren Mock, MS; Brad A Racette, MD; Gary, W. Miller, PhD; Marianthi-Anna Kioumourtzoglou, ScD MSPH; Danielle Braun, PhD; Antonella Zanobetti, PhD; Daniel Mork, PhD

Supplement

Supplement Table 1. Degrees of freedom and knot placement for distributed lag nonlinear models. We allowed 3-5 degrees of freedom for the lag dimension with knots equally spaced on the log scale; and fixed 3 degrees of freedom in the exposure dimension and specified that the center knot be placed at one of the following percentiles: 70th, 75th, 80th, 85th, or 90th. Model selection was performed by AIC.

Supplement Table 2. Lag-specific odds ratios of hospitalization with PD up to 2 days after the day of initial exposure to the 95th and 99th percentile compared to the 50th percentile of the warm season daily maximum heat index distribution, nationwide and by climate.

Supplement Table 3. Cumulative odds ratios of hospitalization with PD after 1 to 3 days of sustained exposure to the 95th and 99th percentile compared to the 50th percentile of the warm season daily maximum heat index distribution after adjusting for daily PM_2.5,_ NO_2_, and O_3_ (ozone) levels, nationwide and by climate.

Supplement Table 4. Sex-specific cumulative odds ratios of hospitalization with PD after 1 to 3 days of sustained exposure to the 99^th^ percentile compared to the 50th percentile of the warm season daily maximum heat index distribution, nationwide and by climate.

Supplement Table 5. Race-specific cumulative odds ratios of hospitalization with PD after 1 to 3 days of sustained exposure to the 99th percentile compared to the 50th percentile of the warm season daily maximum heat index distribution, nationwide.

Supplement Figure 1. Time-lagged risk of hospitalization with PD after sustained exposure to the 99th percentile compared to the 50th percentile of the warm season daily maximum heat index distribution, nationwide.

| **Supplement Table 1. Degrees of freedom and knot placement for distributed lag nonlinear models. We allowed 3-5 degrees of freedom for the lag dimension with knots equally spaced on the log scale; and fixed 3 degrees of freedom in the exposure dimension and specified that the center knot be placed at one of the following percentiles: 70th, 75th, 80th, 85th, or 90th. Model selection was performed by AIC.** | | | | | |
| --- | --- | --- | --- | --- | --- |
|  |  | | | |  |
|  | **Lag degrees of freedom** | | **Exposure quantile knot location** | |  |
|  |  |  |  |  |  |
| Nationwide | 3 |  | 0.7 |  |  |
| Temperate | 3 |  | 0.9 |  |  |
| Continental | 4 |  | 0.7 |  |  |
| Arid | 3 |  | 0.9 |  |  |
| Tropical | 3 |  | 0.7 |  |  |
|  |  |  |  |  |  |

| **Supplement Table 2. Lag-specific odds ratios of hospitalization with PD up to 2 days after the day of initial exposure to 95th and 99th percentile compared to the 50th percentile of the warm season daily maximum heat index distribution, nationwide and by climate.^1^** | | | | | |
| --- | --- | --- | --- | --- | --- |
|  | | | | | |
|  | **Lag day^2^** | **Percentiles of**  **the climate-specific heat index distribution**^3^ | | | |
|  |  | **95th vs. 50th %ile** | | **99th vs. 50th %ile** | |
|  |  |  |  |  |  |
| Nationwide | 1 | 1.009 (1.002, 1.015) | * | 1.010 (1.002, 1.017) | * |
| (All climates | 2 | 1.006 (1.002, 1.011) | * | 1.007 (1.002, 1.013) | * |
| combined) | 3 | 1.004 (1.001, 1.007) | * | 1.005 (1.001, 1.009) | * |
|  |  |  |  |  |  |
| Temperate | 1 | 1.013 (1.003, 1.023) | * | 1.015 (1.003, 1.027) | * |
| climates | 2 | 1.008 (1.001, 1.015) | * | 1.010 (1.001, 1.018) | * |
|  | 3 | 1.004 (0.999, 1.009) |  | 1.005 (0.999, 1.011) |  |
|  |  |  |  |  |  |
| Continental | 1 | 1.002 (0.986, 1.018) |  | 1.001 (0.983, 1.020) |  |
| climates | 2 | 1.005 (0.998, 1.013) |  | 1.006 (0.997, 1.014) |  |
|  | 3 | 1.007 (1.000, 1.015) | * | 1.008 (1.000, 1.017) | * |
|  |  |  |  |  |  |
| Arid | 1 | 1.004 (0.978, 1.031) |  | 1.005 (0.974, 1.037) |  |
| climates | 2 | 1.000 (0.981, 1.019) |  | 1.000 (0.978, 1.022) |  |
|  | 3 | 0.996 (0.983, 1.009) |  | 0.995 (0.980, 1.011) |  |
|  |  |  |  |  |  |
| Tropical | 1 | 1.021 (0.983, 1.060) |  | 1.024 (0.981, 1.069) |  |
| climates | 2 | 1.015 (0.987, 1.043) |  | 1.017 (0.985, 1.049) |  |
|  | 3 | 1.009 (0.989, 1.030) |  | 1.011 (0.988, 1.034) |  |
|  |  |  |  |  |  |

^1^ Warm season includes May-September of each year of the study period, 2000-2016.

^2^ “Lag day” refers to the number of days since exposure to extreme heat. Thus, lag day 0 corresponds to the day of exposure, and lag day 1 refers to the 1 day after the initial day of exposure. The OR of hospitalization with PD on lag day 1 quantifies the increased risk of hospitalization due to extreme heat exposure 1 day earlier.

^3^ Starred (*) cells indicate 95% confidence intervals that do not include the null value. Confidence intervals were not adjusted for multiplicity and should be interpreted appropriately. They are not meant for use in formal hypothesis testing.

| **Supplement Table 3. Cumulative odds ratios of hospitalization with PD after 1 to 3 days of sustained exposure to the 95th and 99th percentile compared to the 50th percentile of the warm season daily maximum heat index distribution after adjusting for daily PM_2.5,_ NO_2_, and O_3_ (ozone) levels, nationwide and by climate.^1,2^** | | | | | |
| --- | --- | --- | --- | --- | --- |
|  | | | | | |
|  | **Days of sustained exposure^3^** | **Percentiles of**  **the climate-specific heat index distribution**^4^ | | | |
|  |  | **95th vs. 50th %ile** | | **99th vs. 50th %ile** | |
|  |  |  |  |  |  |
| Nationwide | 1 | 1.009 (1.002, 1.015) | * | 1.010 (1.002, 1.017) | * |
| (All climates | 2 | 1.015 (1.004, 1.027) | * | 1.017 (1.004, 1.030) | * |
| combined) | 3 | 1.019 (1.005, 1.034) | * | 1.022 (1.005, 1.039) | * |
|  |  |  |  |  |  |
| Temperate | 1 | 1.013 (1.003, 1.023) | * | 1.015 (1.003, 1.027) | * |
| climates | 2 | 1.021 (1.004, 1.038) | * | 1.025 (1.005, 1.045) | * |
|  | 3 | 1.025 (1.004, 1.047) | * | 1.030 (1.004, 1.056) | * |
|  |  |  |  |  |  |
| Continental | 1 | 1.002 (0.986, 1.018) |  | 1.001 (0.983, 1.020) |  |
| climates | 2 | 1.007 (0.985, 1.030) |  | 1.007 (0.981, 1.033) |  |
|  | 3 | 1.014 (0.991, 1.039) |  | 1.015 (0.988, 1.043) |  |
|  |  |  |  |  |  |
| Arid | 1 | 1.004 (0.978, 1.031) |  | 1.005 (0.974, 1.037) |  |
| climates | 2 | 1.004 (0.960, 1.051) |  | 1.005 (0.953, 1.060) |  |
|  | 3 | 1.000 (0.944, 1.059) |  | 1.000 (0.935, 1.070) |  |
|  |  |  |  |  |  |
| Tropical | 1 | 1.021 (0.983, 1.060) |  | 1.024 (0.981, 1.069) |  |
| climates | 2 | 1.036 (0.971, 1.105) |  | 1.041 (0.967, 1.121) |  |
|  | 3 | 1.046 (0.963, 1.136) |  | 1.052 (0.957, 1.157) |  |
|  |  |  |  |  |  |

^1^ These models include additional covariates adjusting for daily ZIP code-specific PM_2.5_, NO_2_, and O_3_ (ozone). Compare these results with those in Table 2, which reports estimates from models that are not adjusted for air pollution.

^2^ Warm season includes May-September of each year of the study period, 2000-2016.

^3^ “Days of sustained exposure” are the number of consecutive days of exposure to the listed heat index. One day of sustained exposure corresponds to lag day 0 in distributed lag models. Because effects of extreme heat persist for up to 2 days past the initial day of exposure, effects accumulate as the duration of exposure increases.

^4^ Starred (*) cells indicate 95% confidence intervals that do not include the null value. Confidence intervals were not adjusted for multiplicity and should be interpreted appropriately. They are not meant for use in formal hypothesis testing.

| **Supplement Table 4. Sex-specific cumulative odds ratios of hospitalization with PD after 1 to 3 days of sustained exposure to the 99th percentile compared to the 50th percentile of the warm season daily maximum heat index distribution, nationwide and by climate.^1,2^** | | | | | |
| --- | --- | --- | --- | --- | --- |
|  | | | | | |
|  | **Days of sustained exposure^2^** |  | | | |
|  |  | **Female** | | **Male** | |
|  |  |  |  |  |  |
| Nationwide | 1 | 1.008 (0.982, 1.034) |  | 1.011 (0.993, 1.029) |  |
| (All climates | 2 | 1.021 (0.996, 1.048) |  | 1.016 (0.991, 1.041) |  |
| combined) | 3 | 1.034 (1.005, 1.062) |  | 1.017 (0.991, 1.043) |  |
|  |  |  |  |  |  |
| Temperate | 1 | 1.027 (1.011, 1.044) |  | 1.004 (0.988, 1.020) |  |
| climates | 2 | 1.048 (1.018, 1.078) |  | 1.004 (0.977, 1.032) |  |
|  | 3 | 1.061 (1.024, 1.100) |  | 1.001 (0.967, 1.037) |  |
|  |  |  |  |  |  |
| Continental | 1 | 0.991 (0.954, 1.030) |  | 1.011 (0.986, 1.037) |  |
| climates | 2 | 0.996 (0.958, 1.036) |  | 1.019 (0.984, 1.056) |  |
|  | 3 | 1.008 (0.966, 1.052) |  | 1.025 (0.987, 1.064) |  |
|  |  |  |  |  |  |
| Arid | 1 | 0.999 (0.956, 1.044) |  | 1.008 (0.966, 1.052) |  |
| climates | 2 | 0.995 (0.923, 1.073) |  | 1.010 (0.940, 1.086) |  |
|  | 3 | 0.989 (0.899, 1.088) |  | 1.006 (0.918, 1.103) |  |
|  |  |  |  |  |  |
| Tropical | 1 | 1.000 (0.937, 1.068) |  | 1.043 (0.983, 1.106) |  |
| climates | 2 | 0.999 (0.892, 1.118) |  | 1.076 (0.972, 1.191) |  |
|  | 3 | 0.996 (0.861, 1.151) |  | 1.099 (0.965, 1.253) |  |
|  |  |  |  |  |  |

^1^ Warm season includes May-September of each year of the study period, 2000-2016.

^2^ “Days of sustained exposure” are the number of consecutive days of exposure to the listed heat index. One day of sustained exposure corresponds to lag day 0 in distributed lag models. Because effects of extreme heat persist for up to 2 days past the initial day of exposure, effects accumulate as the duration of exposure increases.

^3^ Starred (*) cells indicate 95% confidence intervals that do not include the null value. Confidence intervals were not adjusted for multiplicity and should be interpreted appropriately. They are not meant for use in formal hypothesis testing.

| **Supplement Table 5. Race-specific cumulative odds ratios of hospitalization with PD after 1 to 3 days of sustained exposure to the 99th percentile compared to the 50th percentile of the warm season daily maximum heat index distribution, nationwide.^1,2^** | | | |
| --- | --- | --- | --- |
|  |  | | |
|  | **Days of sustained exposure^2^** | | |
|  | **1** | **2** | **3** |
| White | 1.003 (0.984, 1.022) | 1.010 (0.991, 1.029) | 1.015 (0.995, 1.036) |
| Black | 1.031 (0.999, 1.064) | 1.055 (0.999, 1.113) | 1.071 (1.000, 1.148) |
| Hispanic | 1.033 (0.980, 1.089) | 1.057 (0.966, 1.157) | 1.071 (0.955, 1.203) |
| Asian | 1.146 (1.069, 1.230) | 1.259 (1.116, 1.421) | 1.330 (1.140, 1.552) |
| Other | 1.026 (0.950, 1.108) | 1.051 (0.922, 1.199) | 1.075 (0.909, 1.272) |
|  |  |  |  |
| ^1^ Warm season includes May-September of each year of the study period, 2000-2016. | | | |
| ^2^ “Days of sustained exposure” are the number of consecutive days of exposure to the listed heat index. One day of sustained exposure corresponds to lag day 0 in distributed lag models. Because effects of extreme heat persist for up to 2 days past the initial day of exposure, effects accumulate as the duration of exposure increases. | | | |

**Supplement Figure 1. Time-lagged risk of hospitalization with PD after sustained exposure to the 99th percentile compared to the 50th percentile of the warm season daily maximum heat index distribution, nationwide.**
